# Bifurcation analysis of a SEIR epidemic system with governmental action and individual reaction

**DOI:** 10.1186/s13662-020-02997-z

**Published:** 2020-10-01

**Authors:** Abdelhamid Ajbar, Rubayyi T. Alqahtani

**Affiliations:** 1 Department of Chemical Engineering, King Saud University, Riyadh, Saudi Arabia; 2 Department of Mathematics and Statistics, College of Science, Imam Mohammad Ibn Saud Islamic University (IMSIU), Riyadh, Saudi Arabia

**Keywords:** SEIR model, Stability, Bifurcation, Governmental action, Individual response, Hopf bifurcation

## Abstract

In this paper, the dynamical behavior of a SEIR epidemic system that takes into account governmental action and individual reaction is investigated. The transmission rate takes into account the impact of governmental action modeled as a step function while the decreasing contacts among individuals responding to the severity of the pandemic is modeled as a decreasing exponential function. We show that the proposed model is capable of predicting Hopf bifurcation points for a wide range of physically realistic parameters for the COVID-19 disease. In this regard, the model predicts periodic behavior that emanates from one Hopf point. The model also predicts stable oscillations connecting two Hopf points. The effect of the different model parameters on the existence of such periodic behavior is numerically investigated. Useful diagrams are constructed that delineate the range of periodic behavior predicted by the model.

## Introduction

The spread of infectious diseases is a very complex phenomenon that depends on a large number of factors. Some of them are social, environmental, or economic, which are linked to human activities while other factors have to do with the nature of the pathogen causing the disease. With this complexity, it is not surprising that sometimes simple mathematical models are often used to understand the dynamics of spread of infectious diseases. The use of more complex models will necessarily involve very large number of parameters that are difficult to estimate, making the model predictions weak and uncertain. In this regard, compartmental models are often used to simplify the mathematical modeling of infectious diseases. In these models, the population is divided into compartments, with the assumption that every individual in the same compartment has the same characteristics [1]. The models are usually investigated through deterministic ordinary differential equations. The SEIR mathematical model is an extensively used compartmental epidemic model that is based on the division of the population into four basic compartments; an individual can either be susceptible (*S*), exposed to the disease but not yet infectious (*E*), infectious (*I*), or removed (recovered or deceased) (*R*). The SEIR model has been used extensively in the literature to model many human infections such as Ebola [2], H1N1 [3], influenza [4] and MERS-CoV [5]. Despite being an old model, the SEIR model is quite flexible and many variations and improvements on the model are still possible. Right now, the SEIR model has been applied extensively to analyze the COVID-19 pandemic [6–9]. Each of these studies includes a variation on the basic SEIR model by either taking into consideration new variables or parameters, ignoring others, selecting different expressions for the transmission rate, or using different methods for parameter estimation. The stability, bifurcation and chaotic behavior of SEIR epidemic models have been investigated over many decades [10–18]. Tools from nonlinear theory were shown to be useful in revealing conditions for the occurrence of sustained epidemic equilibria. Some early work [10, 11] analyzed the effect of seasonal fluctuations as well as contact rate periodicity in what becomes a forced response problem resulting in harmonic and subharmonic resonances. Several authors have analyzed the occurrence of periodic solutions in SEIR models due to the presence of time delays and/or nonlinear incidence rates [12–16]. Chaotic motion has also been studied in such models [17, 18].

In this paper we examine the bifurcation behavior of a SEIR model when the transmission rate takes explicitly into account governmental action and population response to the severity of the pandemic. Although a number of studies have included the effects of governmental action and individual response in models of COVID-19 [6, 19], there is few work in the literature on the bifurcation and stability of such models. The recent work of Kwuimy et al. [19] showed the importance of governmental action and social behavior in COVID-19 dynamics.

A final note is to be made about the usefulness of such models in the forecast of the spread of COVID-19 pandemic. Because of the absence of complete data for the disease in Saudi Arabia, the validation of the model could not be carried out. However, the values of model parameters that were used in the mathematical analysis were taken from the literature where a validation of a similar model was carried out for the Wuhan province in China [6]. This provides the proposed model with some credibility. Also, one of the objectives of this paper is to carry out a sensitivity analysis for the effect of model parameters. In this regard, the numerical analysis was carried out for a wide range of values of model parameters, which should also give some credibility to the different behavior predicted by the model. Moreover, it is true that some of the behavior predicted by the proposed model (such as periodic solutions), were not reported so far in the current literature on the COVID-19 disease. However, it is also true that the pandemic is unfortunately not yet contained, with the unfortunate possibility of a second wave or more. Given that the proposed model assumes the disease to be endemic and given the long time period involved, it is not ruled out that new data would report cases of some type of periodic behavior.

The rest of this paper is organized as follows. In Sect. 2, we propose the SEIR model with individual reaction and governmental action. A static analysis is carried out in Sect. 3, dynamic analysis in Sect. 4, while numerical simulations are carried out in Sect. 5.

## The dimensional model

We consider the following SEIR model:

1 $$\begin{aligned} &{\frac{\textrm{d}S}{\textrm{d}t}} =b(N-S)-{ \frac{\beta \mathit{S} \mathit{I}}{N}}, \\ \end{aligned}$$

2 $$\begin{aligned} &{\frac{\textrm{d}E}{\textrm{d}t}} ={ \frac{\beta \mathit{S} \mathit{I}}{N}}- ( \alpha +b ) { \mathit{E}}, \end{aligned}$$

3 $$\begin{aligned} &{\frac{\textrm{d}I}{\textrm{d}t}} =\alpha \mathit{E}- ( \gamma +b ) \mathit{I}, \end{aligned}$$

4 $$\begin{aligned} &{\frac{\textrm{d}R}{\textrm{d}t}} =\gamma \mathit{I}-b\mathit{R}, \end{aligned}$$

5 $$\begin{aligned} &{\frac{\textrm{d}P}{\textrm{d}t}} =\mathit{e} \gamma \mathit{I}- \lambda \mathit{P}, \end{aligned}$$

6 $$\begin{aligned} &N =S+E+I+R. \end{aligned}$$

The model equations (Eqs. (1)–(4)) are based on the classical “Susceptible-Exposed-Infectious-Removed” (SEIR) model for a population of size (*N*). The model attempts to describe what may unfortunately be an endemic disease (which may persist in a population for long time). Therefore the vital dynamics (births and natural deaths) have to be incorporated in the model. The variable (*S*) is the fraction of susceptible individuals (those able to contract the disease), (*E*) the fraction of exposed individuals (those who have been infected but are not yet infectious), (*I*) the fraction of infective individuals (those capable of transmitting the disease) and (*R*) is the fraction of removed individuals (those who have recovered or deceased). The model assumes that recovered individuals do not revert to the susceptible class. It is also assumed that all newborns are susceptible with the birth rate set equal to the death rate which is assumed not to be related to the infectious disease.

The term $\frac{\beta I }{N}$ represents the force of infection where *β* is the effective per capita contact rate of infected individuals. The incidence rate is therefore $\frac{\beta I S }{N}$. The parameter *b* is the rate of natural birth, *α* is the rate at which the exposed individuals become infective, so $\frac{1}{\alpha }$ represents the mean latent period. The term $\frac{1}{\gamma }$ represents the mean infectious period. We have added to the classical SEIR model a new equation (Eq. (5)) and a new variable (*P*) that mimics the public perception of the severity of the pandemic. It can be seen that the dynamics (Eq. (5)) of the public perception of the risk regarding the pandemic is proportional to the number of infected cases (*I*) with *e* being the proportion of severe cases and $\frac{1 }{\lambda }$ the mean duration of public reaction.

Besides the new variable (*P*), we adopt in this paper a new expression for the transmission rate *β* that reflects the impact of governmental action and the public perception of the severity of the disease. A number of studies have considered specific forms of the transmission rate. Lin *et al.* [6], for instance, adopted the following expression for the transmission rate which was based on the formulation of He *et al.* [20]:

7 $$\begin{aligned} &\beta =\beta _{0}(1-\mu ) \biggl(1- \frac{D }{N}\biggr)^{\kappa } \end{aligned}$$

where *D* is a state variable representing social behavioral dynamics. The first term in Eq. (7) incorporates the impact of governmental action. It is parameterized by *μ* and represents all actions, which can be modeled as a step function. The second term in *β* represents the decreasing contacts among individuals reacting to the severity of the pandemic. The parameter *κ* represents the intensity of the individual reaction.

Kwuimy *et al.* [19], on the other hand, proposed the following expression:

8 $$\begin{aligned} &(1-\mu ) \bigl(\beta _{1}SI (1-D)^{\kappa } +\beta _{2} SE\bigr). \end{aligned}$$

Similarly to Eq. (7), *D* represents social behavioral dynamics, *μ* represents the strength of the government action and *κ* is the strength of public response. Both expressions (Eqs. (7)–(8)) reflect the fact that public reaction would increase when more people get infected, and would naturally diminish over time. In this paper we assume the following expression for the transmission rate:

9 $$\begin{aligned} &\beta =\beta _{0}(1-\mu )\exp \biggl(\frac{-\kappa P }{N}\biggr). \end{aligned}$$

While keeping the same formulation for the impact of governmental action (all actions which can be modeled as a step function), we have opted for an exponential function to reflect the decreasing contacts among individuals reacting to severity of the disease. Mathematically, the expression $\exp (\frac{-\kappa P}{N})$ can be considered as a good approximation of $(1- \frac{P }{N})^{\kappa }$ (Eq. (7)), especially if the values of *P* are very small compared to the total population *N*, as the numerical simulations will show in this paper.

## Static analysis

The model at steady state has two equilibria: A trivial one ($S=N$, $E=0$, $I=0$, $R=0$, $P=0$) and a nontrivial one, which satisfies the following transcendental equation:

10 $$\begin{aligned} &\beta (\alpha +b) (b+\gamma )I +b\bigl(b(b+\gamma )+\alpha (b-\beta +g) \bigr)N=0. \end{aligned}$$

We have

11 $$\begin{aligned} &\beta =\beta _{0} (1-\mu ) \exp \biggl(\frac{-\kappa e\gamma I }{\lambda N} \biggr). \end{aligned}$$

In the absence of governmental action ($\mu =0$) and public reaction ($\kappa =0$), the transmission coefficient is constant $\beta =\beta _{0}$, and the nontrivial steady state can be solved readily to yield explicit relations for the model state variable *I*:

12 $$\begin{aligned} &I=\frac{ bN(\alpha (-b+\beta _{0}-\gamma )-b(b+\gamma ) }{(\alpha +b) \beta _{0}(b+\gamma )} \end{aligned}$$

The other state variables *S*, *E*, *R* and *P* can be obtained accordingly.

### Positivity of solution

In the following, we show that the model solutions are positive under non-negative initial conditions.

#### Theorem 1

*Let* $S_{0}, E_{0}, I_{0}, R_{0}, P_{0} \geq 0$. *The solution of* (1)*–*(6) *with* $(S(0), E(0), I(0), R(0, P(0)) = (S_{0}, E_{0}, I_{0}, R_{0},P_{0})$, *is non*-*negative*, *that is*, $S(t), E(t), I(t),R(t), P(t) \geq 0$, *for* $t > 0$, *and it satisfies* $S(t) + E(t) + I(t) + R(t) = N$, *with* *N* *constant*.

#### Proof

Let $x(t) = (S(t), E(t), I(t), R(t), P(t))$ be the solution of system under initial conditions $x_{0} = (S(0), E(0), I(0), R(0), P(0)) = (S_{0}, E_{0}, I_{0}, R_{0}, P_{0})\geq 0$.

By continuity of the solution, for all $S(t)$, $E(t)$, $I(t)$, $R(t)$ and $P(t)$ that have a positive initial value at $t = 0$, we have the existence of an interval $(0, t_{0})$ such that $S(t), E(t), I(t), R(t),P(t)\geq 0$ for $0 < t < t_{0}$. We will prove that $t_{0} = \infty $.

If $S(t_{1}) = 0$ for $t_{1}\geq 0$ and other solutions stay positive at $t = t_{1}$, then

13 $$\begin{aligned} &\frac{dS}{dt}(t = t_{1}) = bN. \end{aligned}$$

This implies that whenever the solution $x(t)$ touches the *S*-axis, the derivative of *S* is non-decreasing and the function $S(t)$ does not cross to negative values. Similarly, when $E(t_{1}) = 0$ for a $t_{1} > 0$ and the other solutions stay positive,

14 $$\begin{aligned} &\frac{dE}{dt}(t = t_{1}) = \frac{\beta \mathit{S} \mathit{I}}{N} \geq 0. \end{aligned}$$

When $I(t_{1}) = 0$ for a $t_{1} > 0$ and the other solutions stay positive,

15 $$\begin{aligned} &\frac{dI}{dt}(t = t_{1}) =\alpha \mathit{E}\geq 0, \end{aligned}$$

when $R(t_{1}) = 0$ for a $t_{1} > 0$ and the other solutions stay positive,

16 $$\begin{aligned} &\frac{dR}{dt}(t = t_{1}) =\gamma 1 \mathit{I}\geq 0. \end{aligned}$$

Finally, when $P(t_{1}) = 0$ for a $t_{1} > 0$ and the other solutions stay positive,

17 $$\begin{aligned} &\frac{dP}{dt}(t = t_{1}) =\mathit{e} \gamma 1 \mathit{I} \geq 0. \end{aligned}$$

Therefore, whenever $x(t)$ touches any of the axes $S = 0$, $E = 0$, $I = 0$, $R = 0$, $P=0$, it never crosses them. Now, let $N(t) = S(t) + E(t) + I(t) + R(t)$, we can see that

18 $$\begin{aligned} &\frac{dN}{dt} =0 \end{aligned}$$

so the value of *N* is constant. □

### Stability of trivial solution

Next, we study the stability of the trivial solution. The Jacobian matrix evaluated at $X_{0}(S,E,I,R,P)=(N,0,0,0,0)$ is

$$J(S,E,I,R,P)=\left[\begin{array}{ccccc} -d & 0 & -\beta_{0}(1-\mu ) & 0 & 0\\ 0 & -\alpha -d & \beta_{0}(1-\mu ) & 0 & 0\\ 0 & \alpha & -\gamma -d & 0 & 0\\ 0 & 0 & \gamma & -d & 0\\ 0 & 0 & e\gamma & 0 & -\lambda \end{array}\right].$$

The eigenvalues of the Jacobian matrix $J(S, E, I, R, P )$ evaluated at the disease-free equilibrium are

19 $$\begin{aligned} &\lambda _{1}= -\lambda < 0, \\ &\lambda _{2,3}= -b, \\ &\lambda _{4}= -[\alpha +2 b+\gamma +\sqrt{A}]< 0 , \\ &\lambda _{5}= -[\alpha +2 b+\gamma -\sqrt{A}] , \end{aligned}$$

where

$$\begin{aligned} &A={\alpha }^{2}+ \bigl[ 4[1-\mu ] \beta _{0}-2 \gamma \bigr] \alpha +{\gamma }^{2}. \end{aligned}$$

It follows that the eigenvalues element (Eq. (19)) are negative if

20 $$\begin{aligned} &\beta _{0}< { \frac{ ( \gamma +b ) ( \alpha +b ) }{\alpha ( 1-\mu ) }}. \end{aligned}$$

#### Lemma 1

*The disease*-*free equilibrium* $X_{0}(S,E,I,R,P)=(N,0,0,0,0)$*is locally asymptotically stable provided the condition of Eq*. (20) *is satisfied*.

It should be noted that the results of Eq. (20) can also be derived by obtaining the expression of the basic reproduction number $R_{0}$, which is an important parameter for the monitoring of the spread of the disease but also for the stability of the disease-fee solution. It is known that values of $R_{0}>1$ indicate an unstable equilibrium [21]. The derivation of the expression for $R_{0}$ is carried out in the appendix and yields

21 $$\begin{aligned} &R_{0}=\frac{\alpha \beta _{0}(1-\mu ) }{(\alpha +b)(b+\gamma )}. \end{aligned}$$

The disease-free equilibrium is stable provided that $R_{0} <1$, which is equivalent to Eq. (20).

## Dynamic analysis

In this section we study the conditions of the occurrence of Hopf points in our five-dimensional model. We recall the conditions for a five-dimensional system to exhibit Hopf points [22]. We address the following characteristic equation:

22 $$\begin{aligned} &p(\chi )=\chi ^{5}+b_{1}\chi ^{4}+b_{2} \chi ^{3}+b_{3}\chi ^{2}+b_{4} \chi +b_{5}=0. \end{aligned}$$

This polynomial has exactly one pair of imaginary roots, $\chi _{1,2}=\pm \sqrt{\theta }$, if and only if one of the following sets of conditions is satisfied:

23 $$\begin{aligned} &(C_{1})\quad\Phi =(b_{3}-b_{1}b_{2}) (b_{5}b_{2}-b_{3}b_{4})-(b_{5}-b_{1}b_{4})^{2}=0, \\ &\quad \text{with } \theta =\frac{ (b_{5}-b_{1}b_{4}) }{(b_{3} -b_{1}b_{2}) } >0, \end{aligned}$$

24 $$\begin{aligned} &(C_{2})\quad b_{5}=b_{1}b_{4},\qquad b_{3}=b_{1}b_{2},\quad \text{and}\quad b_{4} < 0, \\ & \quad \text{with } \theta =\frac{1 }{2} \bigl(b_{2}+\sqrt{b_{2}^{2}-4b_{4}} \bigr) >0, \end{aligned}$$

25 $$\begin{aligned} &(C_{3})\quad b_{5}=b_{1}b_{4},\qquad b_{3}=b_{1}b_{2},\quad \text{and}\quad b_{4}=0, \qquad b_{2}>0 \quad \text{with } \theta =b_{2}>0. \end{aligned}$$

The Jacobean *J* of the model is given by

26 $$J(S,E,I,R,P)=\left[\begin{array}{ccccc} -[b+a_{5}] & 0 & -a_{1} & 0 & -a_{2}\\ a_{5} & -a_{3} & a_{1} & 0 & a_{2}\\ 0 & \alpha & -a_{4} & 0 & 0\\ 0 & 0 & \gamma & -b & 0\\ 0 & 0 & e\gamma & 0 & -\lambda \end{array}\right]$$

where

$$\begin{aligned} &a_{0}=\frac{\beta _{0}(1-\mu )}{ N},\qquad a_{1}=a_{0} {\mathit{S}} {{ \mathrm{e}}^{{\frac{\kappa \mathit{P}}{N}}} },\qquad a_{2} = \frac{b\kappa ( N-\mathit{S} ) }{N}, \\ & a_{3}= \alpha +b,\qquad a_{4} =\gamma +b, \\ &a_{5}= a_{0}{\mathit{I}} {\mathrm{e}^{{\frac{\kappa \mathit{P}}{N}}}}. \end{aligned}$$

The coefficients of the characteristic equation of Eq. (22) can be shown to be

27 $$\begin{aligned} &b_{1}= \mathit{I} a y+\mathit{a_{3}}+ \mathit{a_{4}}+2 b+\lambda , \\ &b_{2}= I a y [a_{3}+a_{4}+b+\lambda ]+ [a_{3}+a_{4}] [\lambda +2 b]+2 \lambda b+ {b}^{2} , \end{aligned}$$

28 $$\begin{aligned} &b_{3}= -\Upsilon b + a y I \bigl( {a_{3}} {a_{4}}+ [b+\lambda ] [a_{3}+a_{4}]+b \lambda \bigr) +b \bigl([b+2\lambda ] [a_{3}+a_{4}]+b \lambda \bigr) , \\ &b_{4}= -2\Upsilon {b}^{2} +\lambda ( \mathit{a_{3}}+\mathit{a_{4}} ) {b}^{2}+ a y I \bigl( \mathit{a_{3}} \mathit{a_{4}} [b+\lambda ] + b \lambda [ a_{3}+a_{4}] \bigr), \\ &b_{5}= -\Upsilon {b}^{3}+\mathit{I} ya \mathit{a_{3}} \mathit{a_{4}} \lambda b , \end{aligned}$$

with

29 $$\begin{aligned} &y= \exp \biggl(\frac{-\kappa P}{ N}\biggr)\quad \text{and}\quad \Upsilon ={ \frac{\mathit{e} \gamma \kappa ( N a\alpha y-\mathit{a_{3}} \mathit{a_{4}} )}{N a y}}. \end{aligned}$$

The term Φ in the first Hopf condition $C_{1}$ (Eq. (23)) can be shown to be

30 $$\begin{aligned} &\Phi =(b_{3}-b_{1} b_{2}) (b_{5} b_{2}-b_{3} b_{4})- \bigl((b_{5}-b_{1} b_{4})^{2} \bigr) \\ &\hphantom{\Phi }= f(a,a_{3},a_{4},I,b,y) \kappa + c_{1} c_{2} c_{3} c_{4}, \quad \mbox{with} \end{aligned}$$

31 $$\begin{aligned} &c_{1}=a y I [a_{4}+b] [a_{3}+b]+ 2 b^{2}[a_{3}+a_{4}+b] , \\ &c_{2}= (a y I)^{2}[a_{3}+a_{4}]+ a y I\bigl(a_{3}^{2} +[aa_{3}+a_{4}] [a_{4}+2b]\bigr) + a_{3} b [a_{3}+2 a_{4} + b]+ a_{4} b [a_{4}+b], \\ &c_{3}= a y I [a_{4}+\lambda ] [a_{3}+ \lambda ]+ \lambda [b+\lambda ] [a_{3}+a_{4}+ \lambda ] , \\ &c_{4}= (b+\lambda ). \end{aligned}$$

The obtained expressions for $b_{i}$ ($i=1,5$) and Φ are quite complicated and are not amenable to analytical manipulation; therefore numerical simulations will be carried out in the next section. However, we can obtain an important result for the case when public reaction is absent ($\kappa =0$). In this case it can be seen from Eq. (28) that $\Upsilon =0$ and therefore $b_{4}>0$ (28), which contradicts the Hopf conditions of $C_{2}$ and $C_{3}$ (Eqs. (24)–(25)). As to the first Hopf condition $C_{1}$ (Eq. (23)), it can be seen that for ($\kappa =0$) the term Φ (Eq. (30)) is reduced to $c_{1} c_{2} c_{3} c_{4}$ which is always strictly positive, which contradicts the Hopf condition.

## Numerical simulations

**Figure 1:**
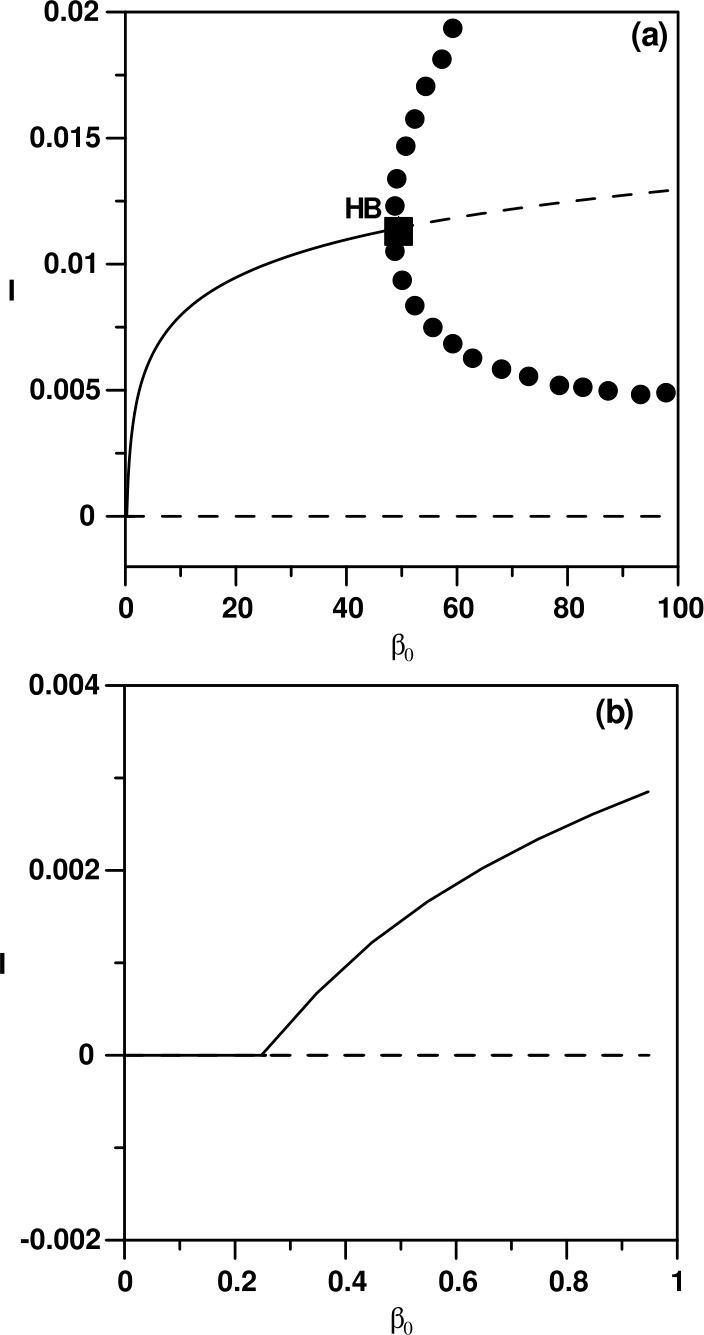
(**a**) Bifurcation diagram when $\beta _{0}$ is the main bifurcation parameter: (**a**) Enlargement of (**a**). (solid) Stable branch; (dash) unstable branch; (filled circle) stable periodic branch; (square) Hopf point

**Figure 2:**
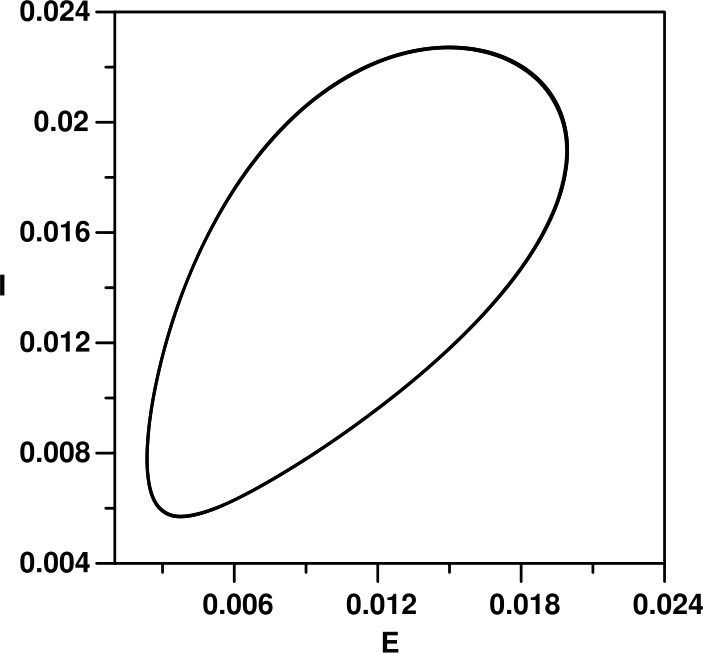
Limit cycle corresponding to Fig. 1 for a value of $\beta _{0}=70$

**Figure 3:**
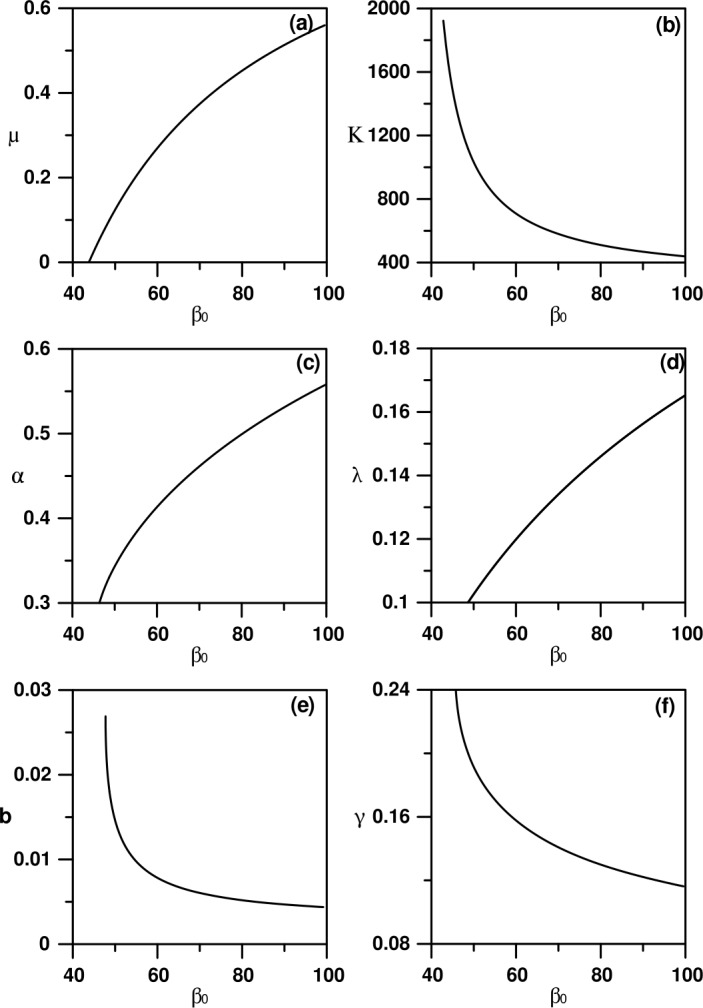
Two parameter continuity diagrams showing the locus of the Hopf point of Fig. 1

The numerical analysis of the model is carried out using standard bifurcation techniques [23] with the help of the software AUTO [24]. The nominal values of the model parameters are listed in Table 1. These parameters correspond to physically realistic values pertinent to COVID-19 disease. Figure 1(a) shows a typical behavior using the transmission rate $\beta _{0}$ as the main bifurcation parameter. There are two static solutions in the diagram: the horizontal trivial solution ($I=0$), and the nontrivial static branch (Eqs. (10)–(11)). The enlargement of the figure shown in Fig. 1(b) indicates that only values of $\beta _{0}$ greater than the value of Eq. (20) will lead to nontrivial solution. The diagram (Fig. 1(a)) is also characterized by the existence of one Hopf point. The periodic branch emanating from the Hopf point can be seen to terminate as it collides with the static branch. For $\beta _{0}$ larger than the value of Eq. (20) and up to the HB point the system will settle on the nontrivial solution, but for $\beta _{0}$ larger than the HB point, periodic behavior is expected in the model for a wide range of $\beta _{0}$. An example of a limit cycle is shown in Fig. 2 for $\beta _{0}=70$. At this point, it is useful to show the effect of the different model parameters on the existence of periodic behavior. Each curve of Fig. 3 shows the locus of the Hopf point. Figure 3(a) shows the effect of governmental action. It can be seen that the model cannot exhibit a Hopf point in the absence of governmental action (i.e. $\mu =0$). Moreover, an increase in the strength of the governmental action (i.e. increase in the value of *μ*), decreases the range of periodic behavior, as the Hopf point moves to larger values of $\beta _{0}$. The effect of the public response (*κ*) on the Hopf point is shown in Fig. 3(b). An increase in the strength of the public reaction (larger *κ*) increases the range of periodic behavior as the Hopf point occurs at small values of $\beta _{0}$.

**Table 1:** Values of model parameters ([6])

Parameter	Notation	Value
Birth rate	*b*	0.018
Proportion of sever cases	*e*	0.2
Mean latent period	$\frac{1 }{\alpha }$	$\frac{1 }{0.33}$
Transmission rate	$\beta _{0}$	1.68
Mean infectious period	$\frac{1 }{\gamma }$	$\frac{1 }{0.2}$
Intensity of responds	*κ*	1117
Mean duration of public reaction	$\frac{1 }{\lambda }$	$\frac{1 }{0.1}$
Strength of government action	*μ*	0.1

The rest of the graphs of Fig. 3 shows that the range of periodic behavior decreases with smaller values of the latent period of the disease (i.e. larger *α*) or smaller values of the mean duration of public reaction (i.e. larger *λ*). On the other hand, the periodic behavior increases with the increase in the birth rate (larger *b*) or we have an increase in the rate of recovery (larger *γ*).

**Figure 4:**
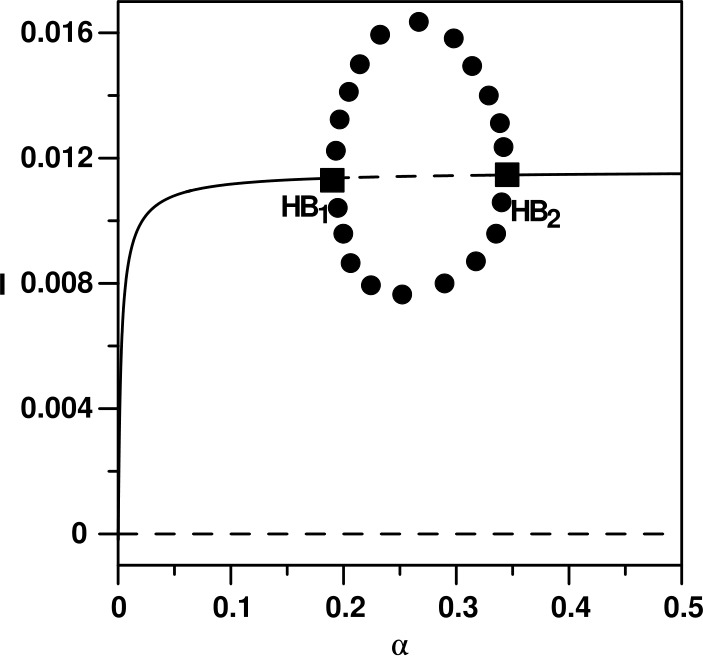
(**a**) Bifurcation diagram when *α* is the main bifurcation parameter. (solid) Stable branch; (dash) unstable branch; (filled circle) stable periodic branch; (square) Hopf point

**Figure 5:**
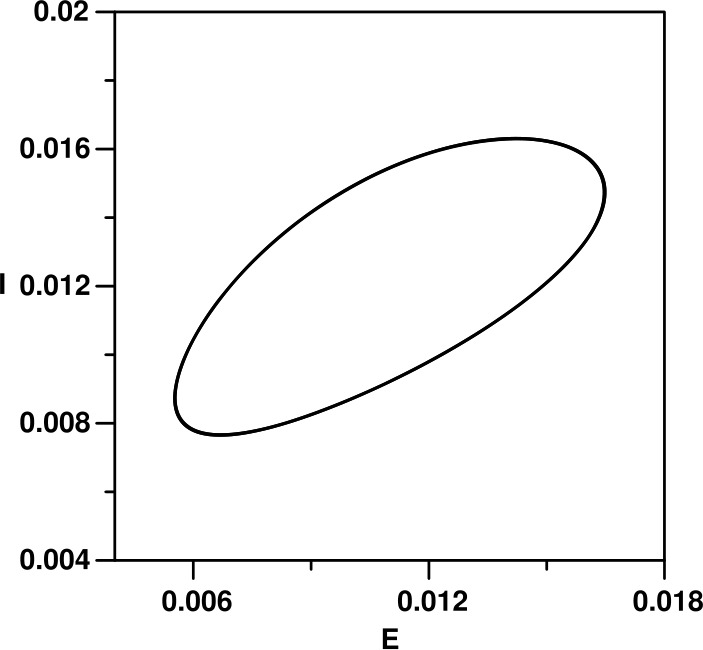
Limit cycle corresponding to Fig. 4 for a value of $\alpha =0.25$

**Figure 6:**
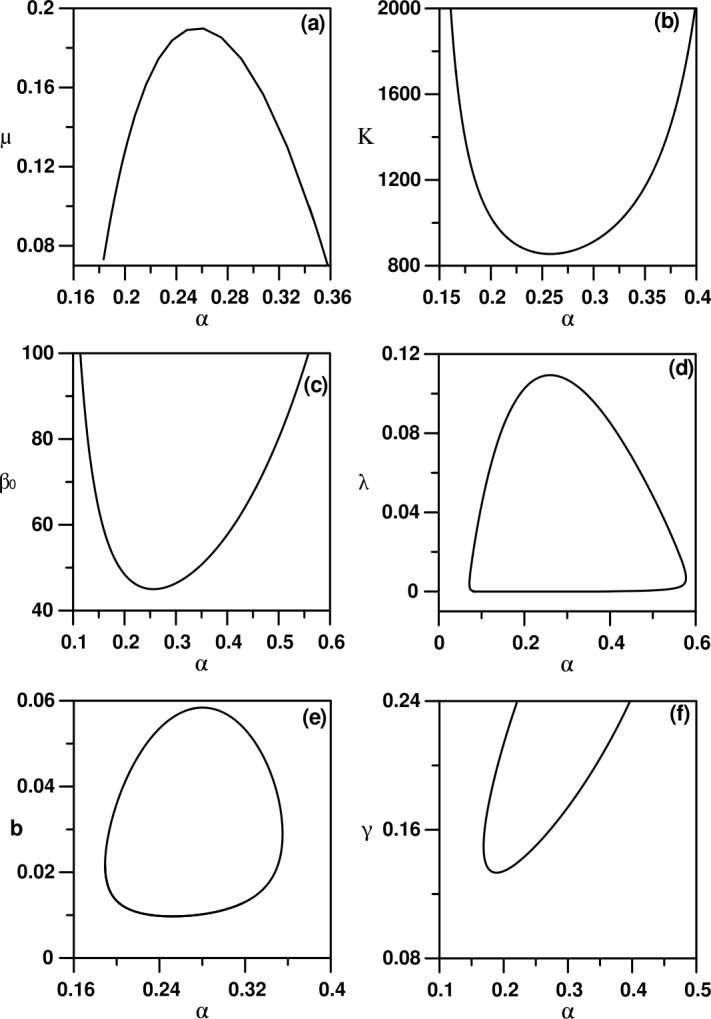
Two parameter continuity diagrams showing the locus of Hopf point of Fig. 4

Another type of bifurcation behavior predicted by the model can be shown in Fig. 4 where *α* (the inverse of the mean latent period) is chosen to be the main bifurcation parameter with the rest of model parameters set at their values of Table 1 with $\beta _{0}=50$. In the diagram the appearance of two Hopf points can be seen that are connected by a stable periodic branch. Unlike the case of Fig. 1, the periodic behavior in this case is confined to a range of values of *α*. Figure 5 shows an example of limit cycle for $\alpha =0.25$. The effect of the different model parameters on the existence of such periodic behavior is shown in Fig. 6 where the two curves show the loci of the two Hopf points. It can be seen that an increase in the strength of the government action (*μ*) decreases the range between the two Hopf points and therefore decreases periodic behavior. The Hopf points will not exist beyond a critical point. On the contrary, increasing the strength of the public reaction (*κ*) increases the range of periodic behavior which will disappear if the strength falls below a critical point. The rest of the curves in Fig. 6 shows that an increase in the basic transmission rate ($\beta _{0}$) would increase the range of periodic behavior, and no periodic behavior can be found below a critical value of $\beta _{0}$. The effect of the mean duration of public reaction shows a closed loop, which means that periodic behavior is confined within two critical values of *λ*. The same can be said about the effect of the birth rate *b*. Periodic behavior is expected only within a specific range of birth rate. Finally, when *γ* increases (i.e. the mean infection periodic decreases) the range of periodic behavior increases. The periodic behavior cannot exist if *γ* falls below a critical value.

## Conclusions

This paper has proposed and analyzed the stability of a SEIR model structured upon susceptible, exposed, infected and removed cases, in addition to a new social behavior variable that mimics the public perception of risk regarding the severity of the pandemic. We also proposed a new expression of the transmission rate that modeled the impact of governmental action as a step function, and the individual reaction as a decreasing exponential function. The model was shown to predict one and two Hopf points. There is a fundamental difference between the two predicted periodic behaviors. While the former would exist for a wide range of model parameters, the latter is generally confined between some critical values. Regardless of the type of periodic behavior (emanating from one or two Hopf points) it was found that periodic behavior will increase in range if the disease has a large latent period, or if the mean duration of the public reaction increases, or the birth rate is high or the rate of recovery increases. Both the governmental action and public reaction have strong effects on the periodic behavior. A periodic behavior would not exist if no governmental action is taken or if there is no individual reaction. The range of periodic behavior would increase with a decrease in the strength of the governmental action or an increase in the strength of the public reaction.

## Appendix: Determination of basic reproduction number

The computation of the basic reproduction [21] number starts by identifying the infected compartments, i.e. *E* and *I*,

32 $$F=\left(\begin{array}{c} \frac{\beta SI}{N}\\ 0 \end{array}\right),$$

33 $$V=\left(\begin{array}{c} (\alpha +b)E\\ -\alpha E+(\gamma +b)I \end{array}\right),$$

where $\mathcal{F}$ denotes the rate of appearance of new infections and $\mathcal{V}$ denotes the rate of transfer of individuals between compartments.

Calculating the derivative of $\mathcal{F}$ and $\mathcal{V}$ with respect to $x = (E, I)$, respectively, then substituting for initial values ($S_{0}$, $E_{0}$, $I_{0}$, $R_{0}$, $P_{0}$) yields

34 $$F=\left(\begin{array}{cc} 0 & \frac{\beta SI}{N}\\ 0 & 0 \end{array}\right),$$

35 $$V=\left(\begin{array}{cc} \alpha +b & 0\\ -\alpha & \gamma +b \end{array}\right).$$

$R_{0}$ is the largest eigenvalue of matrix $FV^{-1}$ i.e. $R_{0} = \rho (FV^{-1})$. Substituting for $\beta =\beta _{0}(1-\mu )\exp (\frac{-\kappa P }{N})$ yields

36 $$\begin{aligned} &R_{0}=\frac{S_{0} \alpha \beta _{0}(1-\mu )\exp (\frac{-\kappa P_{0} }{N}) }{N(\alpha +b)(b+\gamma )} . \end{aligned}$$

Without loss of generality we can take $N=1$. The initial values are $S_{0}=1$ and $P_{0}=1$ for the disease-free equilibrium. This yields

37 $$\begin{aligned} &R_{0}=\frac{\alpha \beta _{0}(1-\mu ) }{(\alpha +b)(b+\gamma )} . \end{aligned}$$
